# Selective Oxidation of Methanol to Methyl Formate on Gold: The Role of Low-Coordinated Sites Revealed by Isothermal Pulsed Molecular Beam Experiments and AIMD Simulations

**DOI:** 10.1021/acs.jpcc.4c03959

**Published:** 2024-08-28

**Authors:** Salma Eltayeb, Lenard L. Carroll, Lukas Dippel, Mersad Mostaghimi, Wiebke Riedel, Lyudmila V. Moskaleva, Thomas Risse

**Affiliations:** † Institut für Chemie und Biochemie, Freie Universität Berlin, Arnimallee 22, 14195 Berlin, Germany; ‡ Department of Chemistry, Faculty of Natural and Agricultural Sciences, University of the Free State, P.O. Box 339, Bloemfontein 9300, South Africa; § Institute of Fundamental Physics, Consejo Superior de Investigaciones Científicas, E-28006 Madrid, Spain; ∥ Institute of Nanotechnology, Karlsruhe Institute of Technology (KIT), P.O. Box 3640, 76021 Karlsruhe, Germany

## Abstract

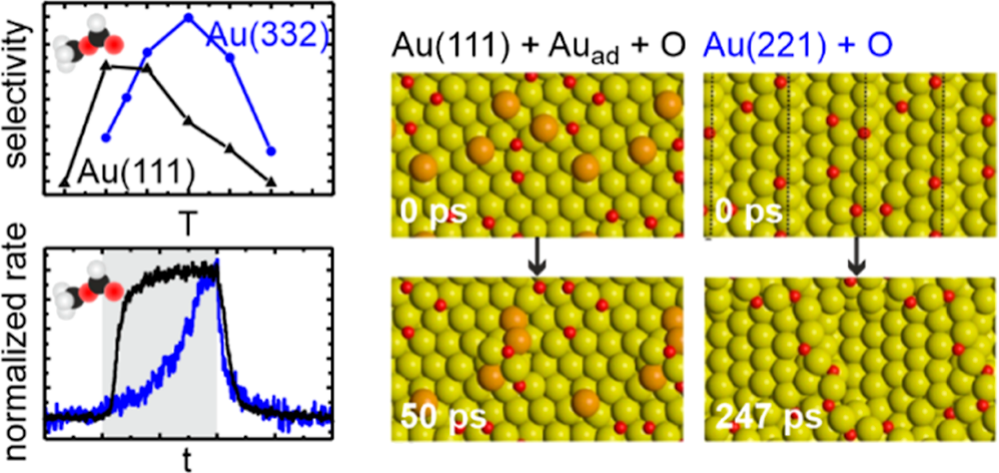

To elucidate the role of low-coordinated sites in the partial methanol oxidation to methyl formate (MeFo), the isothermal reactivity of flat Au(111) and stepped Au(332) in pulsed molecular beam experiments was compared for a broad range of reaction conditions. Low-coordinated step sites were found to enhance MeFo selectivity, especially at low coverage conditions, as found at higher temperatures. The analysis of the transient kinetics provides evidence for the essential role of Au_*x*_O_*y*_ phases for MeFo formation and the complex interplay of different oxygen species for the observed selectivity. Ab initio molecular dynamic simulations yielded microscopic insights in the formation of Au_*x*_O_*y*_ phases on flat and stepped gold surfaces emphasizing the role of low-coordinated sites in their formation. Moreover, associated surface restructuring provides atomic-scale insights which align with the experimentally observed transient kinetics in MeFo formation.

## Introduction

Aerobic partial oxidations are an important class of chemical reactions which may present environmentally friendly substitutes for other synthesis routes. Key for successful partial oxidations are suitable catalysts and a microscopic understanding of the factors governing their selectivity. Methyl formate (MeFo), an essential precursor for various bulk chemicals, exemplifies this, as an alternative to the current industrial synthesis would be desirable, preferably using molecular oxygen.^1−6^ For aerobic methanol oxidation to MeFo, nanoporous gold (npAu)—a sponge-like porous structure of interconnected ligaments with tunable size (usually 10–50 nm)—exhibits a high selectivity at high conversion.^7^ These ligaments not only feature extended terrace sites but also a significant amount of low-coordinated sites, such as steps or kinks.^8^ Residual amounts of the less noble metal (often silver, Ag, from the dealloying of AuAg alloy) play a crucial role in the activation of molecular oxygen enabling thereby its activity in oxidation reactions.^7,9^ In the presence of activated oxygen, the following reaction mechanism for methanol on gold was proposed:^10−12^ First, methanol reacts with oxygen to methoxy species which may yield formaldehyde by reaction with another oxygen. The coupling reaction to methyl formate requires a subsequent reaction of formaldehyde with methoxy and H-abstraction by another oxygen species. However, the intermediately produced formaldehyde may also desorb or undergo overoxidation to formate species finally yielding CO_2_. These pathways compete with MeFo formation. Competing formaldehyde desorption is favored by short contact times, while longer contact times allow for readsorption and subsequent reaction of the formaldehyde contributing to the desired MeFo formation in npAu catalysts under multicollision conditions.^9^ In agreement with expectations, overoxidation is enhanced by high oxygen contents as well as by high amounts of residual Ag allowing for a more efficient activation of molecular oxygen.^7^ Next to the amount of oxygen, also different types of oxygen species, which include distinct accumulated Au_*x*_O_*y*_ phases, affect the selectivity.^10,13−17^ While some factors influencing selectivity have been identified, experimental studies specifically addressing the effect of low-coordinated sites by direct comparison with extended terraces remain scarce,^18^ despite suggestive evidence of reactivity differences.^19−22^

A reason for the limited information on the role of different adsorption sites is the complexity of applied npAu catalysts. Next to various adsorption sites and different types of oxygen species, also multiple-collision conditions within the porous structure contribute to this complexity. However, npAu primarily consists of gold, with only small amounts of residual silver. This composition makes npAu an ideal candidate for comparison with model studies employing single-crystal surfaces under well-defined ultrahigh vacuum (UHV) conditions. While model studies have significantly contributed to the microscopic understanding of this reaction including the proposed reaction mechanism,^10−12^ most model studies exploring methanol oxidation on gold surfaces have employed temperature-programmed reaction (TPR) measurements to investigate reactivity. In contrast, catalytic studies involving npAu studies are typically conducted under isothermal conditions. Recently, pulsed molecular beam (MB) experiments have been used by some of the authors to investigate this reaction under isothermal conditions.^16,20,22^ In these studies, atomic oxygen is directly introduced, as molecular oxygen does not dissociate on gold surfaces under UHV conditions.^23,24^ Our recent comparative study of the isothermal reactivity at 230 K between a flat Au(111) surface and the stepped Au(332) surface highlighted the significance of low-coordinated step sites and the associated oxygen species in mitigating unwanted methanol overoxidation to formate.^18^ Under these specific conditions, the stepped Au(332) surface exhibited increased selectivity toward MeFo.^18^ However, the study focused exclusively on a particular set of reaction conditions, which were notably oxygen-rich, as evidenced by the observed overoxidation. In contrast, oxygen surface concentrations in npAu catalysts operating at ambient pressure are typically very low.^9,25^

In this study, we explore a wide range of reaction conditions in isothermal, pulsed MB experiments on two types of surfaces: a flat Au(111) and a stepped Au(332) surface. Our primary objective is to directly compare the effect of different adsorption sites on the isothermal kinetics governing MeFo formation. The stepped Au(332) surface, characterized by 6-atom-wide (111)-terraces separated by close-packed monatomic steps, exhibits a substantial number of low-coordinated sites alongside close-packed terraces. We systematically vary the surface temperature and also the methanol and oxygen flux (ratio) are changed in the pulsed MB experiments. Thereby, we aim to uncover broader trends related to how extended terraces, as opposed to low-coordinated step sites, influence MeFo formation under isothermal conditions. Furthermore, the analysis of the transient kinetics in these isothermal, pulsed MB experiments provides valuable insights into the behavior of different oxygen species in the partial methanol oxidation, shedding light on the intricate mechanisms at play.

To gain further microscopic insights into surface phenomena on gold surfaces in the presence of oxygen, we have performed ab initio molecular dynamics (AIMD) simulations for the oxygen-covered flat Au(111) and stepped Au(221), which has a structure similar to Au(332) but a narrower terrace width than Au(332). Through these simulations, we gain a deeper understanding of the different surface sites and their interactions with oxygen species. In particular, AIMD sheds light on the intricate surface restructuring processes induced by surface oxygen and into the formation of oxygen phases or aggregates. By dissecting these dynamic phenomena, we illuminate the role of these sites in catalytic processes.

## Experimental and Computational Methods

The measurements were carried out in a UHV apparatus consisting of two chambers maintained at a base pressure of 1 × 10^–10^ mbar, as described previously.^26^ The preparation chamber contains a sputter gun (IQE 11/35, Specs) used for sample cleaning by Ar^+^ ion bombardment, a low-energy electron diffraction (LEED) system (Omicron MCP LEED) to investigate the long-range order of the sample surface, and a quadrupole mass spectrometer (Prisma, Pfeiffer) combined with a Feulner cup for temperature-programmed desorption (TPD) measurements. The scattering chamber is equipped with two effusive MB^27^ for sample exposure to molecular reactants, a thermal oxygen cracker (Dr. Eberl MBE-Komponenten GmbH) providing a well-defined flux of oxygen atoms and a stagnation flow monitor with a high precision ion gauge (360 Stabil-Ion, Granville-Phillips) to monitor the pressure at the sample position. The formation rate of the gas-phase product MeFo (molecular ion H_3_COCHO^+^ at *m*/*z* = 60) in the pulsed isothermal MB experiments was monitored by time-resolved quadrupole mass spectrometric gas-phase analysis (QMS) (MAX–500HT, Extrel). Detection of other gas-phase species, such as formaldehyde and CO_2_, was hindered by overlap with methanol fragmentation and high background reactions in the chamber, respectively. In situ infrared reflection absorption spectroscopy (IRAS) (IFS 66v, Bruker) (256 scans, nominal resolution of 4 cm^–1^, zero filling factor of 16) was used to provide information on surface-adsorbed species present during the reaction.

The Au single crystals (10 mm diameter, 2 mm thick, Mateck) used as model catalysts were pressed with Mo clamps onto a boron nitride heater (HT–01, Momentive) attached to a home-built Mo-holder connected to a liquid nitrogen cooled Cu block, allowing for sample cooling to approximately 100 K. The crystal temperature was measured with a Type K thermocouple inserted into a 0.2 mm hole on the Au crystal edge. A commercial PID controller (3508, Eurotherm) was used to monitor the thermocouple voltage and to control the sample temperature in the isothermal experiments. The Au crystals were cleaned by repeated cycles of Ar^+^ ion bombardment (1.5 × 10^–5^ mbar, ∼6.0 μA, 1 keV) for 15 and 30 min for Au(332) and Au(111), respectively, at room temperature, followed by annealing i. vac. for 10 min at 1000 K for Au(332), and for 10 min at 900 K and 30 min at 700 K for Au(111), until a sharp LEED image expected for the surface was observed.^20,28,29^ Methanol ^12^C (Honeywell Riedel de Haën, Chromasolv, ≥99.9%; dried over molecular sieve, 0.3 Å) was cleaned by repeated freeze–pump–thaw cycles, oxygen (Air Liquide, 99.998%) was used without further cleaning. The thermal cracker (*T* = 1620 °C, 14.15 V, 15.52 A) was employed to supply atomic oxygen to the gold surfaces.

The opening and closing of the effusive beams and the thermal cracker as well as the movement of a nonreactive quartz flag that can be positioned in front of the sample were automated using a custom-made Labview program. The pulse sequence applied in the isothermal MB measurements consists of a long, continuous exposure to a high flux of methanol and one pulse of atomic oxygen. Initially, the quartz flag is positioned in front of the samples, while the methanol beam is started. The quartz flag is moved away from the sample at the beginning of the oxygen pulse (O-pulse) which starts 150 s after the opening of the methanol beam. After the oxygen pulse, which lasted 200 s, the methanol exposure was continued for another 300 s. The methanol flux was calculated according to calibrations with the beam monitor using Ar. The oxygen fluxes were calibrated using TPD measurements on Au(332) and compared to oxygen desorption from Pt(111) precovered with a well-defined amount of oxygen, as evidenced by the formation of a p(2 × 2) superstructure after oxygen exposure at 300 K.^30,31^ The formation rate of MeFo in the chamber was monitored as a function of time (1 s time resolution) using QMS with electron impact ionization (1 mA, 70 eV). The MeFo formation rate was quantified by calibrations applying well-defined pressures of MeFo in the chamber. During the isothermal MB measurements, in situ IRAS measurements were conducted before, during and after the O-pulse using the cleaned Au surface as background.

The AIMD simulations were performed using the CP2K computational package which is known for its computational efficiency.^32^ These simulations allow for studying surface evolution processes as well as the self-assembly of oxygen atoms. In static DFT calculations, CP2K was used to optimize the initial frames used in the AIMD simulations. A p(2 × 6) unit cell was employed for the Au(221) structure, with a slab thickness of approximately 8 Å, together with a vacuum of approximately 10 Å added above the slabs to separate the structure from its periodic counterpart in the *z*-direction. The bottom layer of the Au(221) structure was kept constrained to the bulk geometry, while the top two layers were unconstrained. Lattice parameters for the Au(221) structure of [17.09, 17.09, 18 Å] were used, with cell angles of [90, 90, 90°]. For the Au(111) structure, a p(6 × 6) unit cell (36 Au atoms per layer) was selected for this study, with the lower half of the structure constrained and the upper half left unconstrained. The slab thickness of the Au(111) structure was approximately 9 Å, with a 9 Å vacuum space added on top of the Au(111) structure to separate the structure from its periodic counterpart. Lattice parameters for the Au(111) structure of [17.7, 17.7, 18 Å] were used, with cell angles of [90, 90, 60°]. Six and eight oxygen atoms per unit cell were added to the Au(111) and Au(221) surfaces, respectively, similarly four Au adatoms were added to the Au(111) surface. Oxygen atoms were added to the Au(111) and Au(221) surfaces, as well as Au atoms to the Au(111) surface, pseudorandomly using a custom Python script that employs a convex hull algorithm, triangulation, point generation, and distance analysis. All AIMD simulations in this work used the *NVT* ensemble with the Nosé–Hoover thermostat for temperature control, following previously established practices. A time step of 1 fs was chosen for the simulations, with frames saved every 20 fs. The Perdew, Burke, and Ernzerhof (PBE) functional was used in the calculations,^33,34^ while the Goedecker–Teter–Hutter, PBE (GTH-PBE) pseudopotential,^35^ Gaussian and plane-wave basis sets^36^ were chosen along with a multigrid cutoff energy of 500 Ry. Furthermore, CP2K’s double-ζ basis sets, optimized for GTH pseudopotentials and suitable for both solid and molecular calculations, were used to reduce basis set superposition errors. The Brillouin zone integration was confined to the Γ point for AIMD simulations to reduce computational cost. The convergence threshold for self-consistent electronic minimization was set to 10^–6^ eV. A statistical sampling approach was adopted at an elevated temperature of 700 K to efficiently explore a broad configuration space of surface arrangements. This strategy has previously been shown to be successful in a variety of AIMD studies.^37−39^

## Results and Discussion

### Selectivity of Methyl Formate Formation on Flat Au(111) and Stepped Au(332)

The isothermal partial oxidation of methanol to MeFo was studied on a flat Au(111) surface and a stepped Au(332) surface, by pulsed MB experiments in which a continuous, high flux of methanol and pulses of atomic oxygen (200 s on, 300 s off) were applied. The formation rate of the desired coupling product, methyl formate (MeFo), was monitored by the molecular ion of MeFo (*m*/*z* = 60) using time-resolved mass spectrometry. For the single scattering conditions of the MB experiments, the MeFo selectivity is given as the ratio of the MeFo formation rate to the provided flux of atomic oxygen (O-flux) which were both quantitatively determined taking also into account that two oxygen atoms are required for the formation of one MeFo molecule.

**Figure 1:**
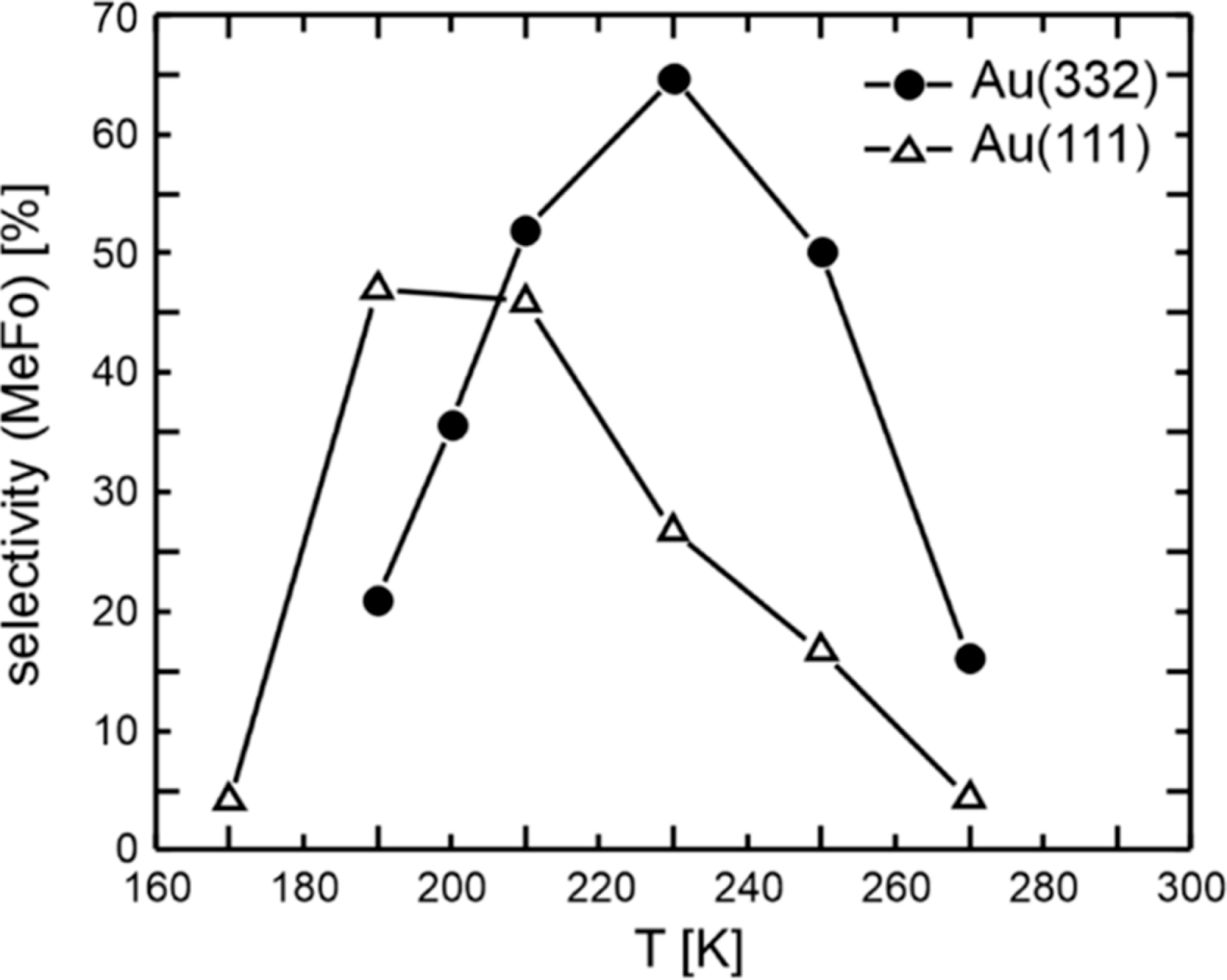
MeFo selectivity at the end of the O-pulse in isothermal, pulsed MB experiments on flat Au(111) (open triangles) and stepped Au(332) (filled circles) as a function of the surface temperature. In these measurements, a methanol flux of 52.7 × 10^13^ s^–1^ cm^–2^ and an O-flux of 0.08 × 10^13^ s^–1^ cm^–2^ were applied.

Figure 1 shows the MeFo selectivity at the end of the O-pulse for different surface temperatures. In these experiments, a methanol flux of 52.7 × 10^13^ s^–1^ cm^–2^ and an O-flux of 0.08 × 10^13^ s^–1^ cm^–2^ were applied, corresponding to a high excess of methanol in the gas phase (factor of approximately 660). For the Au(332) surface, the MeFo rate and selectivity increases, as the surface temperature is raised up to 230 K, reaching a maximum selectivity value of approx 65%. However, beyond this temperature, the MeFo selectivity declined. This behavior can be explained as follows: With increasing temperature, the MeFo formation rate increases due to the temperature-induced enhancement of the rate constant for the rate-limiting step in MeFo formation. Concurrently, higher temperatures accelerate the desorption of reactants and intermediates, such as methanol or formaldehyde, resulting in lower transient surface concentrations. Importantly, the MeFo formation rate depends not only on the rate constant, but also on the surface concentrations of these species. Consequently, a temperature increase may, thus, lower the overall reaction rate, when the surface concentrations of reactants or intermediates decrease significantly. Note that activated oxygen remains adsorbed on the gold surface at all temperatures studied here, desorbing only at elevated temperatures (around 500–550 K).^10,20^

The MeFo selectivity for the flat Au(111) surface (Figure 1) displays a qualitatively similar temperature dependence of the MeFo selectivity as for the stepped Au(332) surface, characterized by a maximum in MeFo selectivity with increasing temperature. However, the MeFo selectivity exhibits a maximum at a lower temperature (around 190 K). Moreover, the maximum MeFo selectivity on flat Au(111) is approximately 47%, which is notably lower than that observed on the stepped Au(332) surface (approximately 65%). Interestingly, at low temperatures (<200 K), the MeFo selectivity is higher on flat Au(111) compared to stepped Au(332). It should be noted that these trends (i.e., the observation of a higher maximum MeFo selectivity at higher temperatures as well as a lower selectivity at low temperatures for stepped Au(332) as compared to flat Au(111)) is not limited to the specific reaction condition discussed here. Similar behavior is found for other oxygen or methanol fluxes (see Figure S1). Thus, these trends are not limited to a specific reaction condition but appear to be a more general feature of the reactivity of these surfaces.

The shift of the maximum to higher temperatures on stepped Au(332) as compared to flat Au(111) and the higher maximum MeFo selectivity can be attributed to higher adsorption energies at low-coordinated sites, such as steps. This rational is corroborated by TPD experiments showing a higher methanol desorption temperature on stepped Au(332) compared to flat Au(111) (see Figure S2). An increased adsorption energy of methanol (or formaldehyde) results in higher local transient surface concentrations at steps compared to extended terraces. Consequently, stepped Au(332) maintains high reaction rates at elevated temperatures, while more rapid desorption on flat Au(111) can significantly lower the surface concentrations of methanol or formaldehyde, thereby impacting the MeFo formation rate. By maintaining higher surface concentrations at elevated temperatures, MeFo formation on stepped Au(332) further benefits from the temperature-dependent increase in the rate constant, resulting in a higher maximum MeFo formation rate and selectivity. Additionally, preferential oxygen adsorption at step sites^19,40^ may further enhance the coupling reaction to MeFo at these low-coordinated sites by increasing the local concentration of this reactant. At low temperatures, slow desorption from the stepped surface can result in a highly covered surface. High coverage conditions impede diffusion of reactants as well as intermediates, lowering the probability of the coupling reaction to MeFo, as a successful coupling reaction requires adsorbed methoxy, formaldehyde and oxygen to encounter each other on the surface and react to MeFo, before unwanted processes, such as formaldehyde desorption, occur. In turn, formaldehyde desorption becomes more likely in case diffusion is hindered. Previously, we observed a lowered MeFo formation and an indication of increased formaldehyde desorption for the Au(332) surface covered with formate species, which can presumably hinder the surface diffusion of other reactants.^20^ While in situ IRAS measurements (Figure S3) provide no evidence for formate accumulation on either Au(332) or Au(111) for the experimental conditions applied in the MB experiments shown in Figure 1, higher transient concentrations of methanol or reaction products, such as MeFo, on the stepped Au(332) surface could also hinder diffusion and thus lower MeFo formation at low temperatures compared to flat Au(111). In agreement, in situ IRAS measurements show at low temperatures notable signals attributable to methanol or methoxy species indicative of rather high transient concentrations, while no signals due to methanol or methoxy are detected at higher temperatures where fast desorption or reactions lower the transient concentrations (Figure S4).

**Figure 2:**
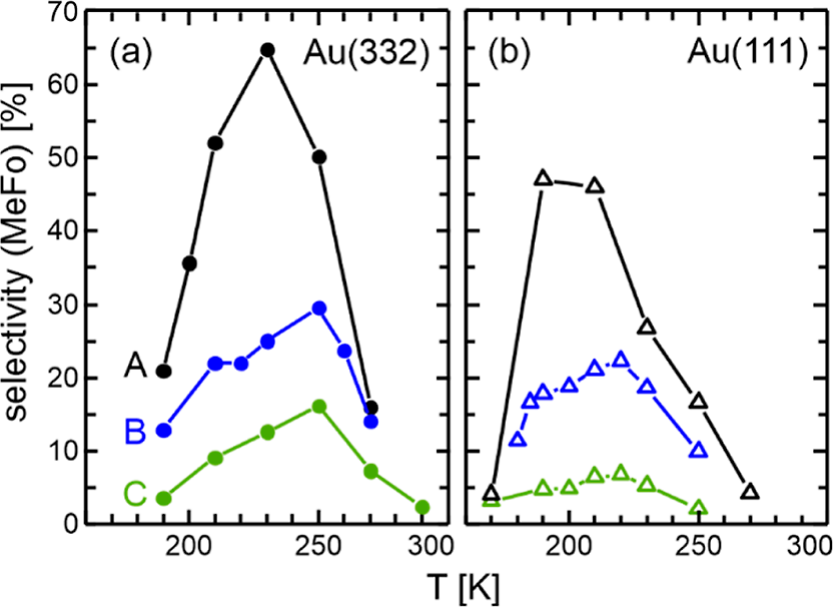
MeFo selectivity at the end of the O-pulse in isothermal, pulsed MB experiments on (a) stepped Au(332) and (b) flat Au(111) as a function of the surface temperature for different methanol and O-fluxes: A (black): methanol flux of 52.7 × 10^13^ s^–1^ cm^–2^ and an O-flux of 0.08 × 10^13^ s^–1^ cm^–2^, B (blue): methanol flux of 52.7 × 10^13^ s^–1^ cm^–2^ and an O-flux of 0.4 × 10^13^ s^–1^ cm^–2^, C (green): methanol flux of 4.3 × 10^13^ s^–1^ cm^–2^ and an O-flux of 0.4 × 10^13^ s^–1^ cm^–2^.

Figure 2 displays the temperature dependence of the MeFo selectivity of Au(111) and Au(332) in partial oxidation of methanol for different methanol and oxygen fluxes varying their ratio by more than a factor of 20. Across all tested conditions, the MeFo selectivity exhibits a maximum with increasing surface temperature. In addition, the MeFo selectivity decreases for both surfaces with increasing oxygen-to-methanol ratios, consistent with previous findings for Au(332) at 230 K.^16^ Concurrently, increasing the oxygen-to-methanol ratio tends to promote unwanted overoxidation. Under the applied experimental conditions, overoxidation can lead to the accumulation of formate species, detectable through in situ IRAS measurements.^16,18,20^ As expected, formate signals appear after the O-pulse for the flat Au(111) surface when a high O-flux is applied, with higher intensity for higher oxygen-to-methanol ratios (see Figure S5). In contrast, for the stepped Au(332) surface, no clear formate signals are detected after a single O-pulse over the entire range of applied reaction conditions. This agrees with previous observations at a surface temperature of 230 K, where formate accumulation rates were lower on the stepped surface a behavior attributed to the formation of Au–O chains at the steps, which exhibit lower reactivity toward overoxidation compared to isolated oxygen atoms.^18^ Interestingly, the absence of clear formate signals after a single O-pulse on the stepped Au(332) surface and significant formate formation on the flat Au(111) surface suggest that low-coordinated step sites can mitigate unwanted overoxidation under various conditions. Thus, step sites play a critical role in determining the selectivity toward the desired partial oxidation product MeFo.

When a higher O-flux is applied, the maximum in MeFo selectivity shifts to higher temperatures for both the flat Au(111) surface (from 190 to 220 K) and the stepped Au(332) surface (from 230 to 250 K). This can be rationalized by assuming that oxygen stabilizes methanol (or formaldehyde) on the surface allowing to maintain MeFo formation also at elevated temperatures. This aligns with reports of increased methanol desorption temperatures from Au(111) precovered with activated oxygen.^11^ This stabilization of reactants or intermediates by oxygen attests to the complex role of oxygen in the methanol oxidation on gold.

### Transient Kinetics in Methyl Formate Formation

**Figure 3:**
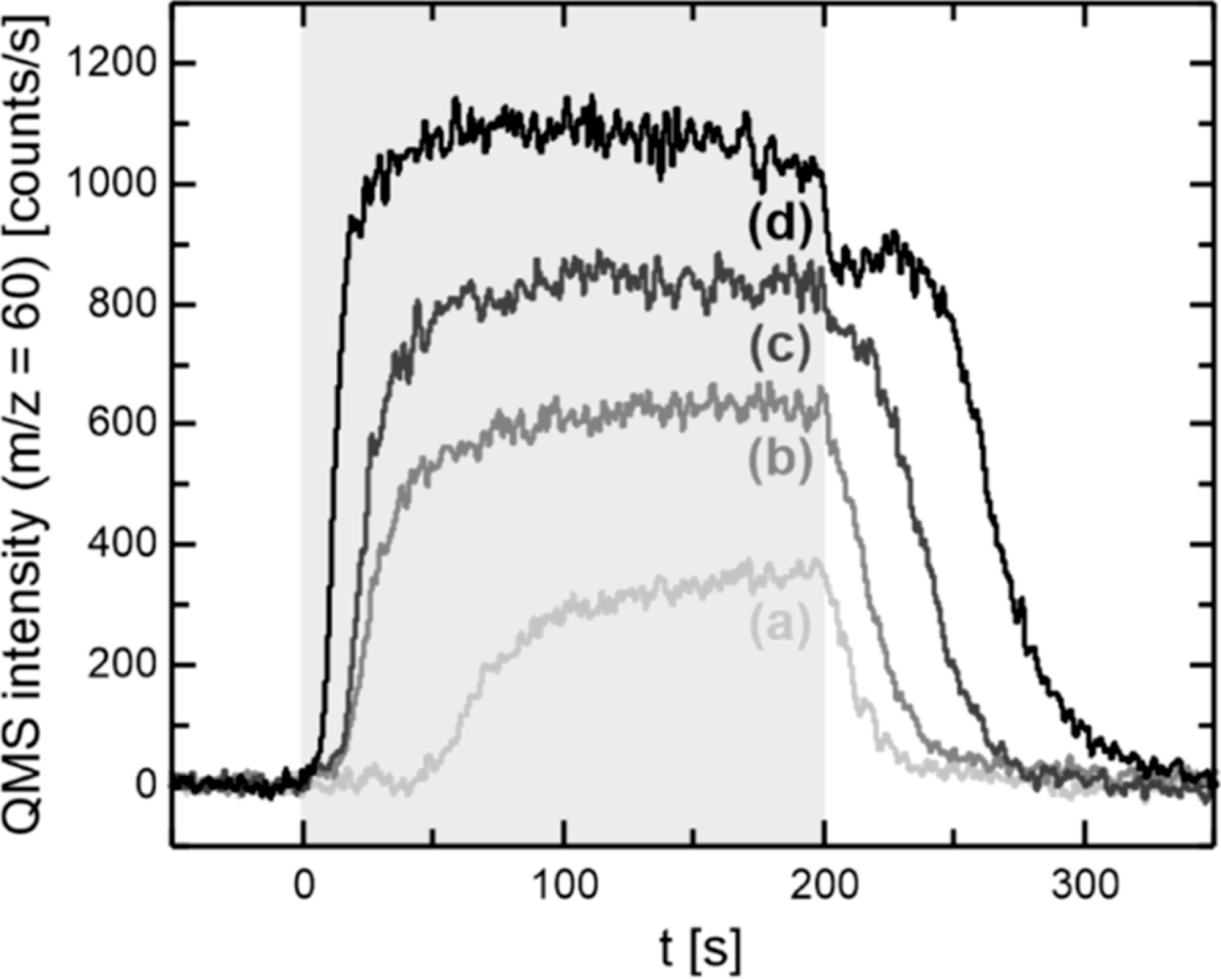
Transient MeFo formation kinetics for Au(111) at a low temperature of 190 K for different O-fluxes: (a) 0.04 × 10^13^ s^–1^ cm^–2^, (b) 0.08 × 10^13^ s^–1^ cm^–2^, (c) 0.17 × 10^13^ s^–1^ cm^–2^, (d) 0.4 × 10^13^ s^–1^ cm^–2^. The methanol flux in all experiments was 52.7 × 10^13^ s^–1^ cm^–2^. The gray shading indicates the duration of the O-pulse.

Further insights into the role of oxygen species are provided by the transient MeFo kinetics measured in the isothermal pulsed MB experiments. Figure 3 exemplarily displays the transient MeFo kinetics at 190 K on Au(111) for varying oxygen fluxes. For small oxygen fluxes, an induction period for MeFo formation is observed. For the lowest applied O-flux (0.04 × 10^13^ s^–1^ cm^–2^), no MeFo is formed immediately after the onset of the O-pulse (*t* = 0 s). MeFo formation starts after about 50 s exhibiting a marked increase of the MeFo formation rate within the next 50 s and a much slower increase until the end of the pulse. As the O-flux increases, the induction period becomes shorter. This decrease in the induction period with increasing O-flux shows that the induction period is connected to the availability of a type of oxygen species on the surface. Moreover, these results are inconsistent with MeFo formation by reaction with isolated oxygen atoms, as only these are available directly after the onset of the oxygen pulse (at *t* = 0 s). The onset of MeFo formation after about 50 s for the lowest oxygen flux clearly indicates that the state of the surface must have changed during the oxygen pulse and that the change is due to oxygen accumulation. The accumulation of oxygen on the surface is readily seen from the reactivity after the oxygen pulse, as will be discussed below. It is known from experiments that oxygen tends to aggregate into Au_*x*_O_*y*_ phases on the (111)-surface.^17,41,42^ However, the formation of these Au_*x*_O_*y*_ phases takes time, which of course depends on the availability of oxygen atoms and hence the oxygen flux. In turn, their formation takes longer at low O-fluxes and becomes faster at higher O-fluxes. In other words, if the accumulation of Au_*x*_O_*y*_ phases is necessary for MeFo formation, an initial induction period may occur, which is expected to decrease with increasing O-flux, in agreement with the experimental results. While the feasibility of MeFo formation with Au_*x*_O_*y*_ phases, as formed by ozone exposure at 200 K, has been previously shown by TPR measurements,^10^ the transient kinetics of the isothermal MB experiments not only support the feasibility of the MeFo formation with accumulated Au_*x*_O_*y*_ phases, but also highlight that MeFo formation using isolated oxygen atoms alone is not readily possible on Au(111) surfaces, which cannot be inferred from the TPR studies, as single oxygen atoms are not stable on the Au(111) surface, but form Au_*x*_O_*y*_ phases already at low temperatures.^17,41,42^

Additional insights can be gained by examining the MeFo formation rate after the end of the O-pulse (*t* > 200 s). First, significant MeFo formation continues even after the end of the O-pulse with increasing MeFo formation at higher O-fluxes. Since the coupling reaction to MeFo relies on oxygen, the ongoing MeFo formation demonstrates that not all oxygen is consumed during the O-pulse through reactions with methanol. Instead, some oxygen remains unreacted, which is expected to reside within the Au_*x*_O_*y*_ phases on the surface.^17,42^ For a constant methanol flux, the amount of unreacted, residual oxygen should increase for higher O-fluxes, leading to more MeFo formation after the O-pulse. Apart from this qualitative consideration, the selectivity toward MeFo was found to drop significantly for increased O-flux, which allows for a higher amount of unreacted oxygen during the O-pulse (see also Figure S6). While this observation agrees qualitatively with expectations, it is clear that oxygen may also be consumed by reactions competing with MeFo formation, such as formaldehyde desorption or CO_2_ formation. Unfortunately, a quantification of these reaction rates by QMS is not possible under the conditions used in the isothermal MB experiments due to large fragmentation signals from methanol or methyl formate as well as background signals in the UHV chamber. Attempts to quantify the residual oxygen coverage by TPD failed due to the large amount of methanol accumulating on the sample holder during the MB experiment hampering proper TPD measurements of desorbing oxygen due to coinciding methanol desorption from different parts of the sample holder.

Although it was not possible to quantitatively analyze the residual amount of oxygen present on the surface after the O-pulse, and consequently to quantify how much of this residual oxygen is consumed by MeFo formation (after the end of the O-pulse), we are able to gain further insight into the role of accumulated Au_*x*_O_*y*_ phases on Au(111) in MeFo formation by examining the temporal evolution of MeFo formation after the O-pulse in more detail. At the highest applied oxygen flux, MeFo formation initially drops, but then increases slightly before decreasing again. This kinetic behavior requires different reactivities of the oxygen within the accumulated Au_*x*_O_*y*_ phases. If all oxygen atoms had the same reactivity, the MeFo rate should decrease monotonically as the surface concentration of the Au_*x*_O_*y*_ phases decreases. Hence, the observed increase in reaction rate toward MeFo in the absence of oxygen flux is evidence for a heterogeneous ensemble of sites with different reactivities toward MeFo formation. This is consistent with TPR results showing a decreasing selectivity toward MeFo formation with increasing size of the Au_*x*_O_*y*_ phases.^17^ Upon reaction with methanol, the size of Au_*x*_O_*y*_ phases will decrease, which can cause an increasing rate of MeFo formation despite an overall decreasing oxygen concentration.

While no clear increase in the MeFo formation rate after the end of the pulse is observed for lower O-fluxes on Au(111) at 190 K, the rate decrease is clearly not a simple exponential decay, as expected for a system with only one type of oxygen species. The rate decrease shortly after the end of the O-pulse is slow but accelerates with time before decelerating again. For lower O-fluxes, a lower amount of residual oxygen is expected, resulting in smaller Au_*x*_O_*y*_ phases. Smaller islands were found to react more rapidly,^17^ consequently, the MeFo rate will drop faster after the O-pulse for smaller O-fluxes in line with the experimental results.

**Figure 4:**
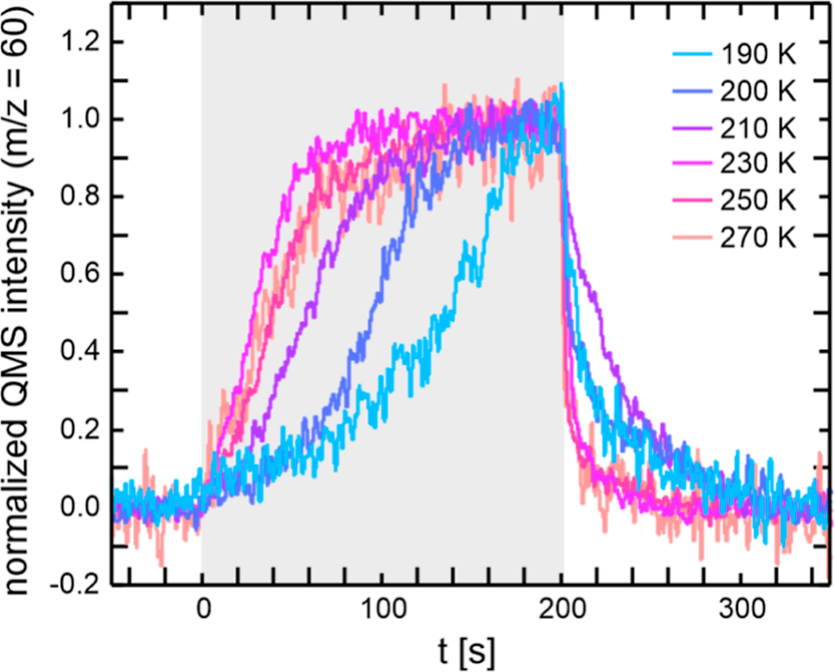
Transient MeFo formation kinetics for Au(332) at a rather low O-flux of 0.08 × 10^13^ s^–1^ cm^–2^ as a function of surface temperature. A methanol flux of 52.7 × 10^13^ s^–1^ cm^–2^ was applied in these experiments. The traces are normalized to the MeFo rate at the end of the O-pulse to allow for a direct comparison of the transient kinetic behavior. The gray shading indicates the duration of the O-pulse.

Figure 4 presents the transient MeFo formation kinetics for the stepped Au(332) surface as a function of surface temperature using a low oxygen flux (*f*(O) = 0.08 × 10^13^ s^–1^ cm^–2^) and a high methanol to oxygen flux ratio of 660 (*f*(MeOH) = 52.7 × 10^13^ s^–1^ cm^–2^). Please note that the data is normalized to the MeFo rate at the end of the O-pulse to allow for a better comparison of the transient behavior. Similar to the results shown in Figure 3 for flat Au(111), an induction period is also observed for the stepped Au(332) at low temperatures. This suggests that MeFo formation on the stepped surface also involves some kind of accumulated Au_*x*_O_*y*_ phase. As the surface temperature increases, the induction period becomes shorter. This phenomenon could be attributed to the expected faster formation of Au_*x*_O_*y*_ phases at higher temperatures, facilitated by accelerated surface diffusion of oxygen being an activated process.^43−45^ In this respect, it is important to note that oxygen tends to accumulate at the step edges^19,37,40,46^ and recent AIMD simulations have also shown that oxygen atoms adsorbed on a stepped surface can cause a rapid restructuring of the surface.^18^ Consequently, the induction period for MeFo formation should decrease at higher temperatures, in agreement with the experimental results.

MeFo formation after the end of the O-pulse (*t* > 200 s) can also be observed on the stepped Au(332) surface. For the applied methanol and oxygen fluxes, MeFo formation following the O-pulse increases with increasing temperature from 190 to 210 K, but then decreases again for temperatures ≥230 K. It is interesting to note that the transient decay at 210 K exhibits a clear bimodal behavior with a very fast drop of MeFo formation rate directly after the end of the pulse followed by a prolonged MeFo formation. Thus, for temperatures ≤210 K, increasing the temperature allows to utilize more residual oxygen, which was not consumed during the O-pulse, for MeFo formation presumably by increasing the rate constant of the rate-limiting step in MeFo formation. Considering that the MeFo formation also increases with temperature during the O pulse, while the amount of oxygen supplied remains constant, suggests that a significant amount of oxygen remains unreacted on the surface at low temperatures (note that the rate constants of competing reactions consuming oxygen are also expected to increase at higher temperatures). As the temperature continues to increase above 210 K, the MeFo formation after the O-pulse decreases and the MeFo rate drops quickly (see also Figure S7). If competing pathways for consumption of oxygen would be neglected, smaller oxygen consumption during the O-pulse at elevated temperatures (>230 K, see also Figures 1 and S7) should result in more residual oxygen and thus, more MeFo formation after the pulse. Thus, MeFo formation after the pulse might even extend to longer times compared to lower temperatures, as oxygen consumption will take longer, when the transient concentrations of methanol or formaldehyde become lower due to fast desorption at elevated temperatures. As this is clearly not observed, it is more likely that oxygen is consumed by a competing reaction. For the applied low oxygen flux of 0.08 × 10^13^ s^–1^ cm^–2^, the formation and desorption of formaldehyde is expected to dominate oxygen consumption rather than overoxidation.

When comparing the results at 190 K for an oxygen flux of 0.08 × 10^13^ s^–1^ cm^–2^ for the flat Au(111) surface and the stepped Au(332) surface (with the same high methanol flux of 52.7 × 10^13^ s^–1^ cm^–2^), an induction period is observed for both surfaces under these conditions. However, the induction periods differ significantly between the stepped Au(332) and the flat Au(111) surfaces. The behavior of the stepped Au(332) surface is different in the sense that MeFo formation starts almost immediately after the oxygen pulse is switched on. However, the rate of MeFo formation increases rather slowly for more than 100 s, before flattening off after a steeper increase at about 160 s which is significantly longer than observed on the (111) surface where this flattening off is observed around 100 s after the start of the pulse (Figure 3). The lack of a clear induction period on Au(332) aligns with both experimental and theoretical findings, which suggest that oxygen atoms at step sites are likely to play an important role for MeFo formation on the stepped surface. We expect a rapid assembly of oxygen atoms at the step edges facilitated by the relatively short diffusion length for oxygen atoms on the (111) terraces. However, it is less obvious why it takes even longer to reach the maximum rate of MeFo formation on the stepped Au(332) surface than on the Au(111) surface. The accumulation of oxygen on the surface during the pulse, coupled with the reduced selectivity toward MeFo, makes the scarcity of available oxygen an improbable factor. Given the preferential adsorption of oxygen at step sites and the short diffusion length, one could logically anticipate an even higher probability for the formation of accumulated Au_*x*_O_*y*_ phases as the reaction progresses. Therefore, it appears that “ordinary” oxygen atoms adsorbed at the step edges may not be particularly well suited for MeFo formation. Rather, it is probable that subsequent processes, such as the oxygen-induced surface restructuring, play a significant role. This hypothesis is consistent with the previously inferred notion of a diverse ensemble of surface oxygen species, among which the most active for MeFo formation will take time to form at low temperature.^18^

### Oxygen-Induced Restructuring of Gold Surfaces

**Figure 5:**
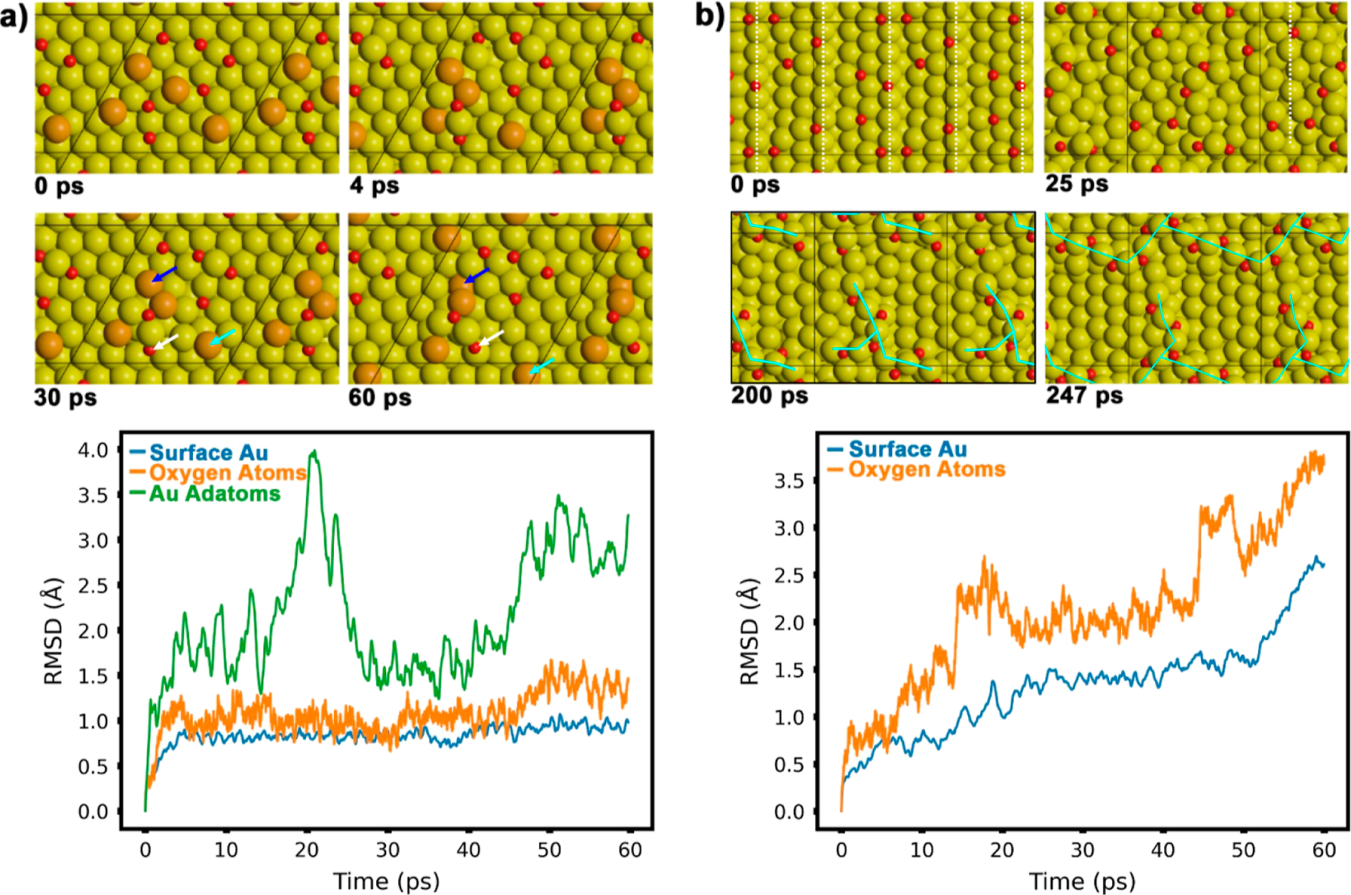
Snapshots from AIMD simulations illustrating different degree of surface restructuring induced by adsorbed atomic oxygen (top) and root-mean-square displacements (RMSD) of the different types of surface atoms (bottom) on (a) flat Au(111) with Au adatoms (oxygen coverage: 0.19 ML) and (b) stepped Au(221) (oxygen coverage: 0.17 ML). Dashed white lines in the initial snapshot (0 ps) indicate the position of the step edges. Color coding: Au, yellow; O, red; Au adatoms [Au(111) surface], orange.

To gain a deeper insight into the surface processes that occur upon oxygen exposure, we performed AIMD simulations at 700 K (Figure 5). This temperature was selected after a preliminary set of short test simulations at different temperatures (300, 700, and 1000 K), which indicated that 700 K provided sufficient mobility at least on the stepped Au surface for examining the restructuring of the stepped surface. Our investigation compared a flat and a stepped gold surface—both oxygen covered—to determine the significance of the step sites in surface restructuring. The flat Au(111) and the stepped Au(221) surfaces were analyzed, with the latter closely resembling the experimentally used Au(332) surface characterized by (111) terraces and close-packed steps but with a narrower terrace width than Au(332) thus reducing computational demands (see Figure S8).

The Au(111) surface model incorporated Au adatoms, anticipated to emerge on the surface after lifting of the herringbone reconstruction induced by oxygen adsorption. Notably, the two surfaces exhibited marked differences in terms of the amount of restructuring during the AIMD simulations. For the Au(111) surface, the RMSD of the Au atoms (blue trace, Figure 5a, bottom) remained minimal throughout the simulation, indicating very little structural change. The constant RMSD value below 1 Å found for these atoms suggests primarily vibrational motion within their potential wells. The RMSD values of the oxygen atoms followed a similar trend, with an initial rise followed by stabilization until approximately 45 ps (orange trace). A subsequent slight increase in RMSD can be attributed to the movement of one of the six oxygen atoms present in the unit cell (indicated by white arrows in the snapshots at 30 and 60 ps, Figure 5a).

In contrast, the Au adatoms (highlighted in orange) displayed significantly greater mobility. The increase of RMSD values above about 45 ps is largely associated with the diffusion of Au adatoms (see cyan arrows in the snapshots at 30 and 60 ps, Figure 5a). In addition, an exchange process between adatoms and surface atoms is observed (dark blue arrows in the snapshots at 30 and 60 ps, Figure 5a), a process well-known from the literature also for close-packed metal surfaces.^47−49^ While this implies that oxygen atoms may not be required for such an exchange process, it is found that surface oxygen atoms in a 3-fold coordination induces lateral stress which pushes surface Au atoms outward which in turn may facilitate such processes. While four of the six oxygen atoms remain stable on or close to the original 3-fold hollow sites (as positioned by the DFT calculations used to generate the starting configuration), two participate in the formation of a small gold cluster in which they are arranged in a short linear chain motif, O–Au–O. It is important to note that the linear OAuO species is distinguished from the ordinary oxygen atoms adsorbed on the surface by the fact that the Au atoms in the structure are lifted out of the surface layer. The cluster begins to form as early as 4 ps around one of the two OAuO species and involves the Au adatoms responsible for the fluctuation during the simulation. As mentioned earlier, one of the three Au adatoms attached to the structure is incorporated into the surface after about 50 ps, resulting in the Au_4_O_2_ cluster. The formation of such Au_*x*_O_*y*_ clusters is linked to the presence of Au adatoms on the surface. Figure S9 shows two sets of AIMD simulations with two different concentrations of oxygen atoms on the Au(111) surface. In both simulations, the majority of the oxygen atoms remain in their 3-fold hollow adsorption sites, and those that change position adopt a similar local coordination after the diffusion event. Again, linear OAuO species form in some instances (when two O atoms were placed near the same Au atom in the initial structure), but no significant movement of O atoms or surface restructuring beyond small displacements was found. These results suggest that Au adatoms play an important role in cluster formation, presumably stabilized by the presence of oxygen atoms. Consistent with this, a previous STM study on an oxygen-covered Au(111) surface reported the formation of disordered Au_*x*_O_*y*_ clusters and suggested that their formation is related to Au adatoms from a lifted herringbone reconstruction.^42^

The situation is considerably different for the oxygen-covered stepped Au(221) surface (Figure 5b, oxygen coverage corresponds to 0.17 ML). While the RMSD values for oxygen and surface Au atoms remain stable after an initial raise on the Au(111) surface, the corresponding traces for the (221) surface (Figure 5b, bottom) show a parallel increase of the RMSD values for both atom types, indicating a structural rearrangement starting from the very beginning of the simulation. This is confirmed by the snapshot taken shortly after the start of the simulation. Initially, the surface is characterized by well-defined close-packed steps as indicated by the vertical dashed lines. However, after just 25 ps, these steps are no longer identifiable due to the rearrangement of both gold and oxygen atoms. This observation is consistent with LEED experiments on Au(332),^26^ which show that exposure to oxygen atoms leads to a loss of superstructure spots and a disappearance of diffraction spots well below oxygen saturation coverage. At the beginning of the simulation, half of the O atoms were located at step edges, with all but one adsorbed in 3-fold adsorption sites, as DFT calculations predict these to be one of the favorable adsorption sites. In the snapshot taken after 25 ps, all oxygen atoms are adsorbed close to low-coordinated Au atoms. Together with the Au cluster on the (111) surface formed by the nucleation of Au adatoms near oxygen atoms, this result provides further evidence for the ability of oxygen atoms to stabilize Au atoms with a considerably lower coordination than found on the close-packed (111) terrace. For extended simulation times, further restructuring of the surface is observed. This is evident from the behavior of the RMSD values (Figure S10a). The structural rearrangements lead to the formation of extended chain-like Au–O structures. The presence of an extended Au–O chain structure is clearly seen e.g. in the snapshot taken after 200 ps (cyan traces), and importantly, this structure continues to grow, resulting in an almost completely interconnected network after 247 ps (cyan traces, Figure 5b).

The results clearly demonstrate that the restructuring processes differ between the flat (111) and the stepped (221) surfaces. While oxygen induces restructuring on both Au(111) with Au adatoms and stepped Au(221), the extent of the Au–O structures formed due to the increased mobility of surface atoms, is significantly larger on the (221) surface. Previously reported DFT calculations have shown that the reactivity of oxygen species depends on their local environment.^18^ In particular, single oxygen atoms on terraces and terminal oxygen atoms in short chains associated with low-coordinated sites show comparable barriers to overoxidation, whereas nonterminal oxygen atoms within longer Au–O chains show a significantly higher activation barrier to overoxidation. The ability of the stepped surface to rapidly form extended oxygen structures is thus considered beneficial for suppressing overoxidation. Furthermore, the transient kinetics of MeFo formation presented here provide clear evidence that MeFo formation preferentially occurs at sites formed during oxygen pulses. Therefore, the differences in restructuring dynamics, combined with the different reactivity of oxygen species depending on their location and local environment, can explain the different selectivity for MeFo formation observed in the MB experiments on the two surfaces.

### Discussion

The presented results from pulsed isothermal MB experiments on methanol oxidation to MeFo on Au(111) and Au(332), together with AIMD simulations on the restructuring of the oxygen-covered Au surfaces, have significant implications for aerobic methanol oxidation using applied npAu catalysts. First, the combination of extended terraces and low-coordinated sites in npAu appears to be crucial for the observed high selectivity toward MeFo formation. Extended terraces sustain MeFo formation at low temperatures or, more generally, high coverage conditions, thus, where the overall transient concentration of adsorbates is high. Such high coverage conditions are expected for e.g. liquid phase methanol oxidation on npAu. In contrast, low-coordinated steps allow for high MeFo selectivities at higher temperatures or low coverage conditions, as expected for typical reaction conditions applied in gas phase methanol oxidation on npAu. Reactants preferentially adsorb at these low-coordinated sites, resulting in increased local concentrations and, consequently, higher formation rates of the coupling product MeFo, which is especially beneficial under low coverage conditions. In combination, extended terraces and low-coordinated step sites contribute to the observed high MeFo selectivity over a wide range of reaction conditions, as experimentally observed for npAu catalysts.^7^ Second, our results indicate that MeFo formation may critically depend on surface restructuring, resulting in Au_*x*_O_*y*_ phases that differ between extended terraces and low-coordinated steps. This dependence on Au_*x*_O_*y*_ formation helps to better understand the effect of ozone pretreatment of npAu which has been found beneficial in achieving active npAu catalysts.^14^ On one hand, the ozone pretreatment enhances the surface concentration of Ag, which is crucial for molecular oxygen activation and hence for the npAu catalyst activity.^14^ On the other hand, we have recently proposed that the oxidation of stepped Au surfaces results in the formation of Au–O chains at low-coordinated step sites, which can help mitigating unwanted overoxidation.^18^ The results presented here further suggest that the ozone pretreatment may also enhance MeFo formation by facilitating the formation of Au_*x*_O_*y*_ phases and/or by aiding in oxygen-induced restructuring of the surface. As the oxygen surface concentration on npAu is typically low,^9,25^ surface restructuring requiring oxygen for Au_*x*_O_*y*_ phase formation may proceed very slowly in the absence of ozone-pretreatment, which creates a highly oxidized surface. To this end it is important to keep in mind that most of the oxygen deposited during the ozone treatment leads to overoxidation of methanol.^14^ However, after an initial phase with poor selectivity toward MeFo in which excess oxygen is consumed, a steady state activity with high selectivity toward MeFo is reached. In such a steady state, activated oxygen in Au_*x*_O_*y*_ phases is consumed and replenished at equal rates, allowing to achieve and maintain high selectivity toward the desired partial oxidation product MeFo.

While the formation of different types of Au_*x*_O_*y*_ species on gold has been previously reported,^13,14,42^ the current results from the isothermal, pulsed MB experiments clearly emphasize their kinetic heterogeneity in methanol oxidation to MeFo. The transient kinetics of MeFo formation not only indicate differences in the nature of Au_*x*_O_*y*_ phases for stepped Au(332) and flat Au(111), but also reveal that each of the surfaces exhibits several types of oxygen species, also within Au_*x*_O_*y*_ phases, that contribute to the formation of this partial oxidation product. The experimental results provide evidence that MeFo formation is linked to the formation of Au_*x*_O_*y*_ phases. In addition, the results of the AIMD simulations not only provide evidence for the importance of low-coordinated Au atoms for surface restructuring in the presence of oxygen, but also highlight the structural heterogeneity and dynamics of Au_*x*_O_*y*_ structures that are formed. It is thus concluded that the complex dynamics of the structural rearrangements is intimately related to the ability of Au surfaces to perform highly selective partial oxidation of methanol. This result contrasts with typical expectations based on a more simplified reaction mechanism that assumes only one type of homogeneously distributed oxygen species, neglecting the heterogeneity of oxygen species and adsorption sites in gold catalysts. Therefore, this isothermal MB study carried out under well-defined conditions in combination with AIMD simulations contributes significantly to the microscopic understanding of the complex reaction network governing the selectivity in the oxidation of methanol on gold catalysts.

## Conclusions

Pulsed isothermal MB measurements were conducted to study the methanol oxidation on flat Au(111) and stepped Au(332) surfaces in a range of reaction conditions varying not only the surface temperature, but also the methanol and oxygen fluxes.

Flat Au(111) exhibited enhanced MeFo selectivity at low temperatures, while the Au(332) surface with a large number of low-coordinated step sites, allowed for higher MeFo formation at elevated temperatures. The observed reactivity differences can be partially attributed to the preferential adsorption of reactants at these low-coordinated step sites, leading to increased local surface concentrations and consequently high formation rates of the coupling product MeFo under low coverage conditions, as expected at higher temperatures.

The results also highlight the complex role of oxygen in the oxidation of methanol to MeFo: While increasing the oxygen-to-methanol flux ratio lowers MeFo selectivity, high oxygen fluxes also appear to stabilize the reactants at higher temperatures on the surface. Furthermore, the transient kinetics in the pulsed MB experiments provide evidence that MeFo formation may require or at least benefit from the presence of Au_*x*_O_*y*_ phases with a high local oxygen concentration. Notably, the results are clearly inconsistent with a reaction to MeFo occurring only with isolated oxygen atoms. Additionally, the transient kinetics of MeFo formation attest to the kinetic heterogeneity of these Au_*x*_O_*y*_ phases. The oxygen species differ not only for flat Au(111) and stepped Au(332), but also on one surface the Au_*x*_O_*y*_ species vary in their reactivity.

AIMD simulations revealed surface restructuring in the presence of oxygen on both a stepped Au(221) surface and an Au(111) surface with Au adatoms, resulting in distinct Au_*x*_O_*y*_ structures. Assuming that efficient MeFo formation requires the creation of such Au_*x*_O_*y*_ structures through activated surface restructuring in the presence of oxygen, this observation may explain the induction period observed at low temperatures and low oxygen fluxes. Moreover, the presence of different Au_*x*_O_*y*_ structures on the two surfaces aligns with differences in their reactivity.

Consequently, the study, which combines isothermal MB experiments under well-defined conditions with AIMD simulations, significantly enhances the microscopic understanding of low-coordinated sites and various oxygen species within the complex reaction network governing the selectivity to methyl formate in methanol oxidation on gold catalysts.

## Data Availability

Raw and meta data are available under DOI: 10.5281/zenodo.13374233.

## Supporting Information Available

Initial methyl formate selectivity for various reaction conditions on Au(111) and Au(332) in isothermal MB experiments, TPD measurements of methanol on Au(111) and Au(332), in situ IRAS spectra and intensity of formate signals during methanol oxidation on Au(111) and Au(332), integrated *m*/*z* = 60 QMS intensity for methanol oxidation on Au(111) as a function of the oxygen flux, (non-normalized) transient kinetics for methyl formate formation on Au(332), AIMD simulations for oxygen on Au(111) without Au-adatoms with different oxygen coverage, AIMD simulations on Au(111) with Au-adatoms and Au(221) and corresponding RMSD plots, RMSD plots for Au(221) evaluated for different oxygen species (PDF)
