# Supplementary material for: Selective Oxidation of Methanol to Methyl Formate on Gold: The Role of Low-Coordinated Sites Revealed by Isothermal Pulsed Molecular Beam Experiments and AIMD Simulations
Source: J Phys Chem C Nanomater Interfaces. 2024 Aug 28;128(36):14978–88. doi: 10.1021/acs.jpcc.4c03959 (PMC11403654; doi:10.1021/acs.jpcc.4c03959)
Supplement: Supplementary file 1 — jp4c03959_si_001.pdf [file jp4c03959_si_001.pdf]

**Supporting Information**

# Selective Oxidation of Methanol to Methyl Formate on Gold: The Role of Low-Coordinated Sites Revealed by Isothermal Pulsed Molecular Beam Experiments and AIMD Simulations

Salma Eltayeb,[a] Lenard L. Carroll,[b,c] Lukas Dippel,[a] Mersad Mostaghimi,[b,d] Wiebke Riedel,[a] Lyudmila V. Moskaleva,[b] Thomas Risse[a],*

[a] Institut für Chemie und Biochemie, Freie Universität Berlin, Arnimallee 22, 14195 Berlin, Germany

[b] Department of Chemistry, Faculty of Natural and Agricultural Sciences, University of the Free State, PO Box 339, Bloemfontein 9300, South Africa

[c] Institute of Fundamental Physics, Consejo Superior de Investigaciones Científicas, E-28006 Madrid, Spain

[d] Institute of Nanotechnology, Karlsruhe Institute of Technology (KIT), P.O. Box 3640, 76021 Karlsruhe, Germany

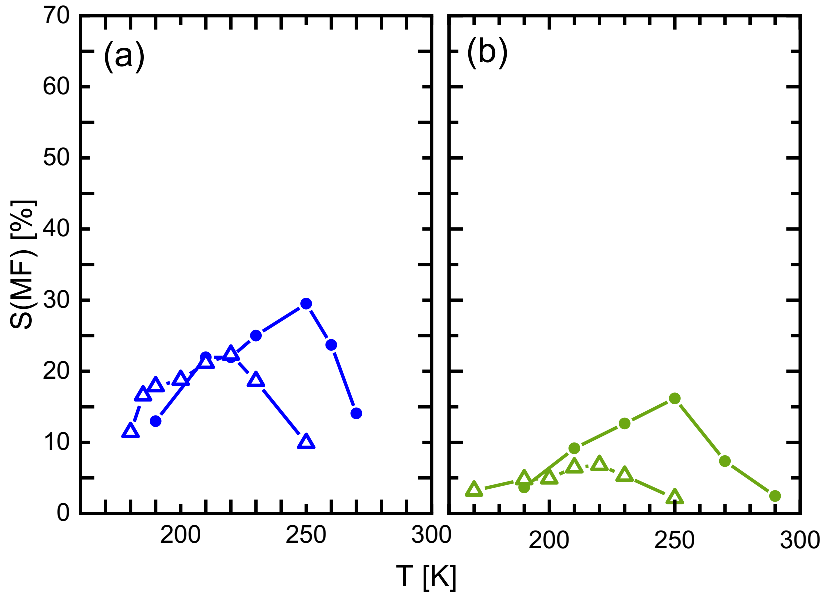


**Figure S1** MeFo selectivity at the end of the O-pulse in isothermal pulsed MB experiments on flat Au(111) (open triangles) and stepped Au(332) (closed circles) for an O-flux of $0.4 \cdot 10^{13}$ $s^{-1}$ $cm^{-2}$ and a methanol flux of (a) $52.7 \cdot 10^{13}$ $s^{-1}$ $cm^{-2}$ and (b) $4.3 \cdot 10^{13}$ $s^{-1}$ $cm^{-2}$.

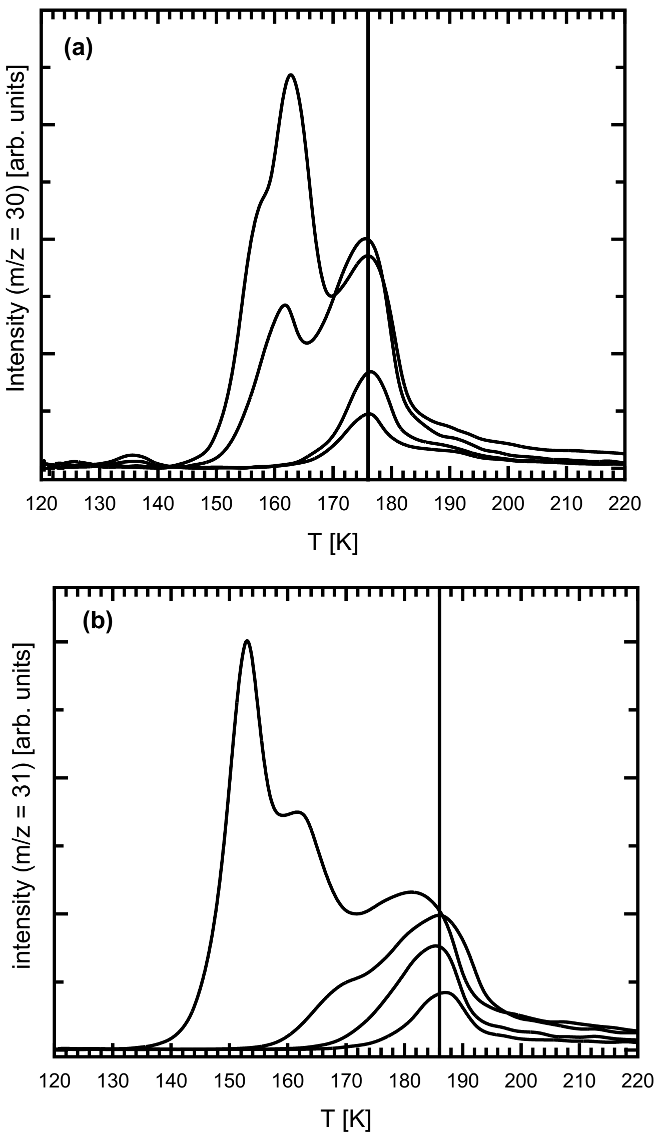


**Figure S2** TPD measurements for different exposures of methanol from (a) the flat Au(111) surface and (b) the stepped Au(332) surface. The spectra were measured at a constant heating rate of 2 K/s. Monolayer desorption of methanol shifted by approx. 10 K to higher temperatures for the stepped Au(332) surface as compared to the flat Au(111) surface.

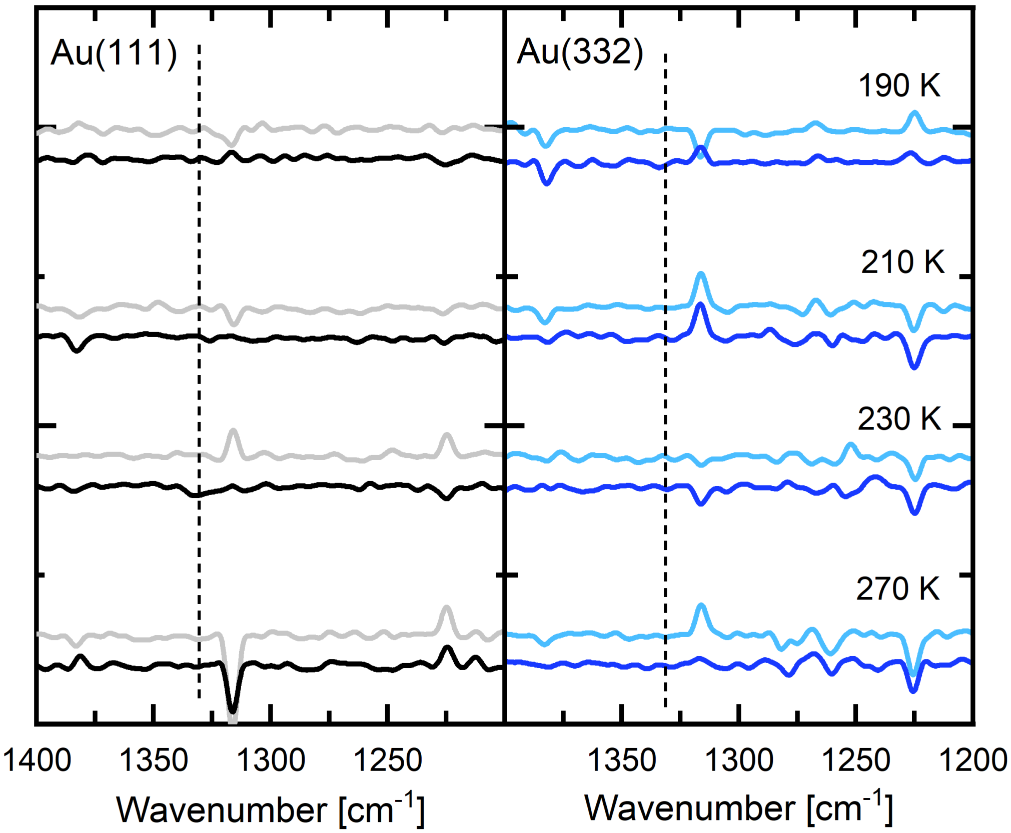


**Figure S3** In-situ IRAS measurements during the isothermal, pulsed MB experiments of the methanol oxidation at 190 - 270 K on Au(111) (left) and Au(332) (right). Vertical dashed line shows the position of a signal characteristic for formate species on the gold surfaces. Gray and light blue spectra were taken during the O-pulse; black and dark blue spectra were taken after the O-pulse. During the experiment a continuous methanol flux of $52.7 \cdot 10^{13}$ $s^{-1}$ $cm^{-2}$ was applied and atomic oxygen was pulsed using a flux of $0.08 \cdot 10^{13}$ $s^{-1}$ $cm^{-2}$ (pulse: 200 s on, 300 s off).

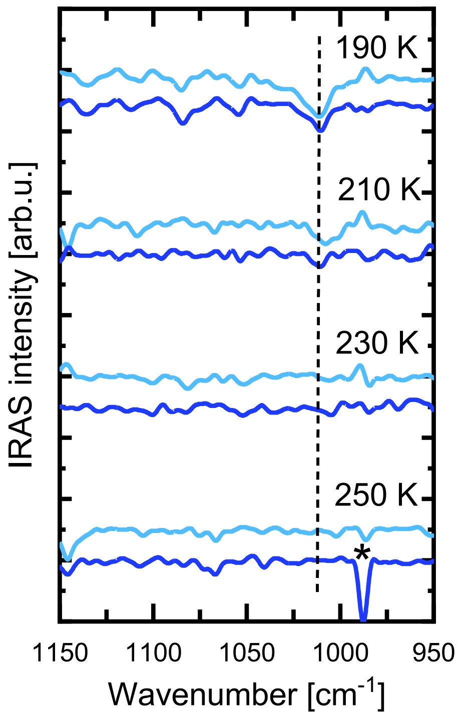


**Figure S4** In-situ IRAS measurements in the wavenumber range between 950 $cm^{-1}$ and 1150 $cm^{-1}$ conducted during the isothermal, pulsed MB experiments of the methanol oxidation in the temperature range between 190 and 250 K on Au(332). Under methanol rich condition (a continuous methanol flux of $52.7 \cdot 10^{13}$ $s^{-1}$ $cm^{-2}$ was applied, while atomic oxygen was pulsed ($0.08 \cdot 10^{13}$ $s^{-1}$ $cm^{-2}$, 200 s on, 300 s off). light blue and dark blue during O-pulse on and O-pulse off, respectively. The peaks around 990 $cm^{-1}$ (indicated for the upper most spectrum with stars) is experimental artefacts.

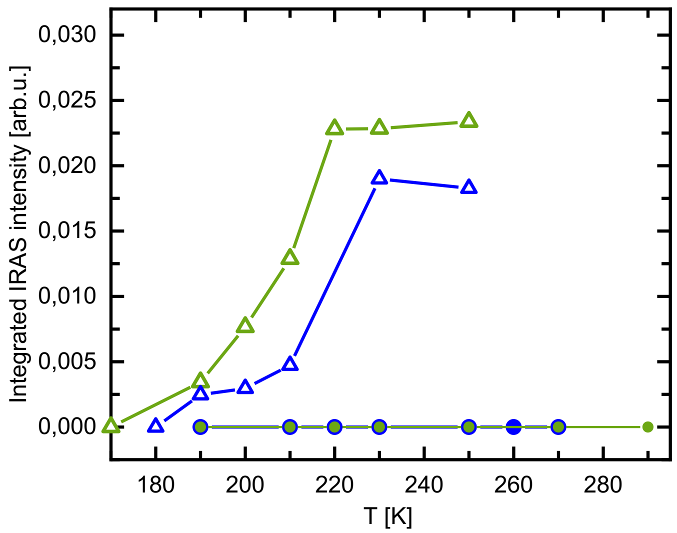


**Figure S5** Integrated IRAS intensity of the OCO-stretching vibration of formate for the flat Au(111) surface (open triangles) and the stepped Au(332) surface (closed circles) after the O-pulse in isothermal pulsed MB experiments applying an O-flux of $0.4 \cdot 10^{13}$ $s^{-1}$ $cm^{-2}$ and a methanol flux of $52.7 \cdot 10^{13}$ $s^{-1}$ $cm^{-2}$ (blue) or $4.3 \cdot 10^{13}$ $s^{-1}$ $cm^{-2}$ (green).

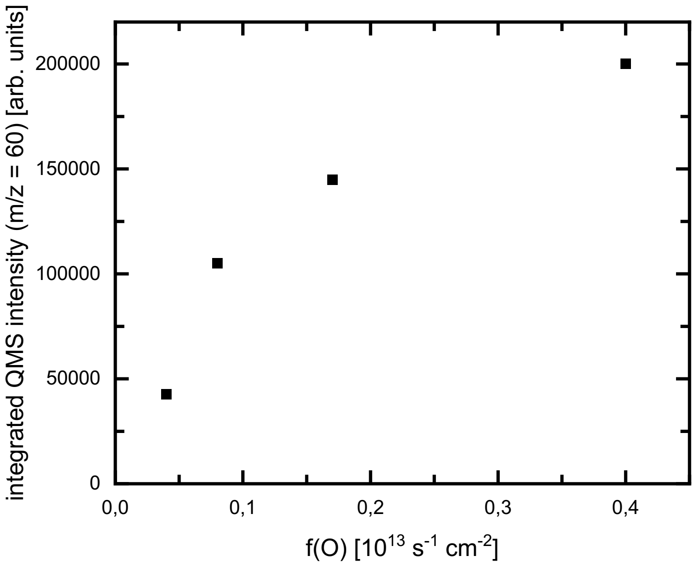


**Figure S6** Integrated QMS intensity of m/z = 60 as a function of the oxygen flux f(O) for methanol oxidation on Au(111) at 190 K applying a methanol flux of $2.7 \cdot 10^{13}$ $s^{-1}$ $cm^{-2}$. The QMS intensity was integrated over the duration of the O-pulse (0-200 s) giving a measure for the amount of formed methyl formate during the O-pulse. The transient kinetic is shown in Figure 3. It can be seen that the amount of formed MeFo is not proportional to the O-flux over the entire range, but that the increase in formed MeFo becomes smaller for higher oxygen fluxes.

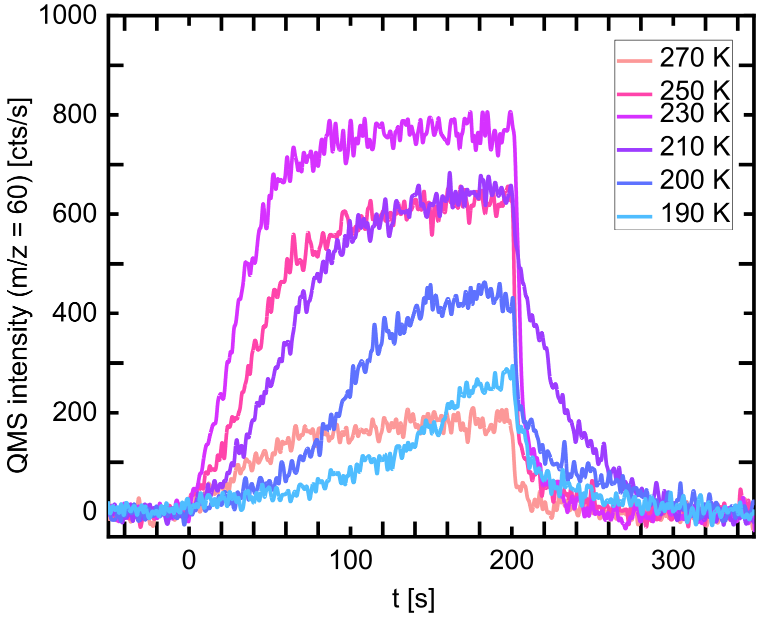


**Figure S7** Transient MeFo formation kinetics for Au(332) at a rather low O-flux of $0.08 \cdot 10^{13}$ $s^{-1}$ $cm^{-2}$ as a function of surface temperature. A methanol flux of $52.7 \cdot 10^{13}$ $s^{-1}$ $cm^{-2}$ was applied in these experiments. In contrast to Figure 4, here the traces are shown without normalization to signal height at the end of the pulse.

a)

b)

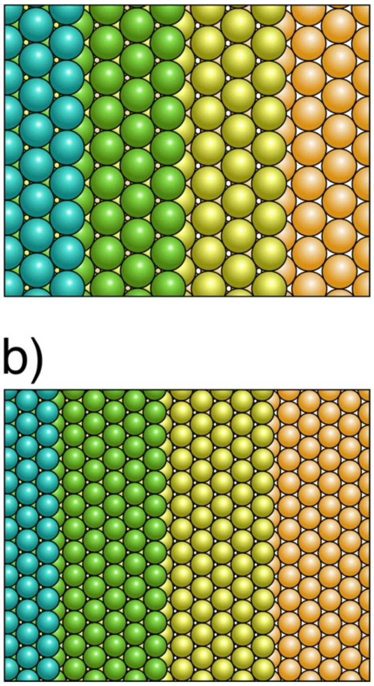

**Figure S8** Structure of an ideal Au(221) (a) and Au(332) (b) surface. Both surfaces exhibit (111) terraces and closed packed steps and differ only in the size of the (111) terraces being 4 and 6 atoms along the closed packed direction on the (221) and the (332) surface, respectively.

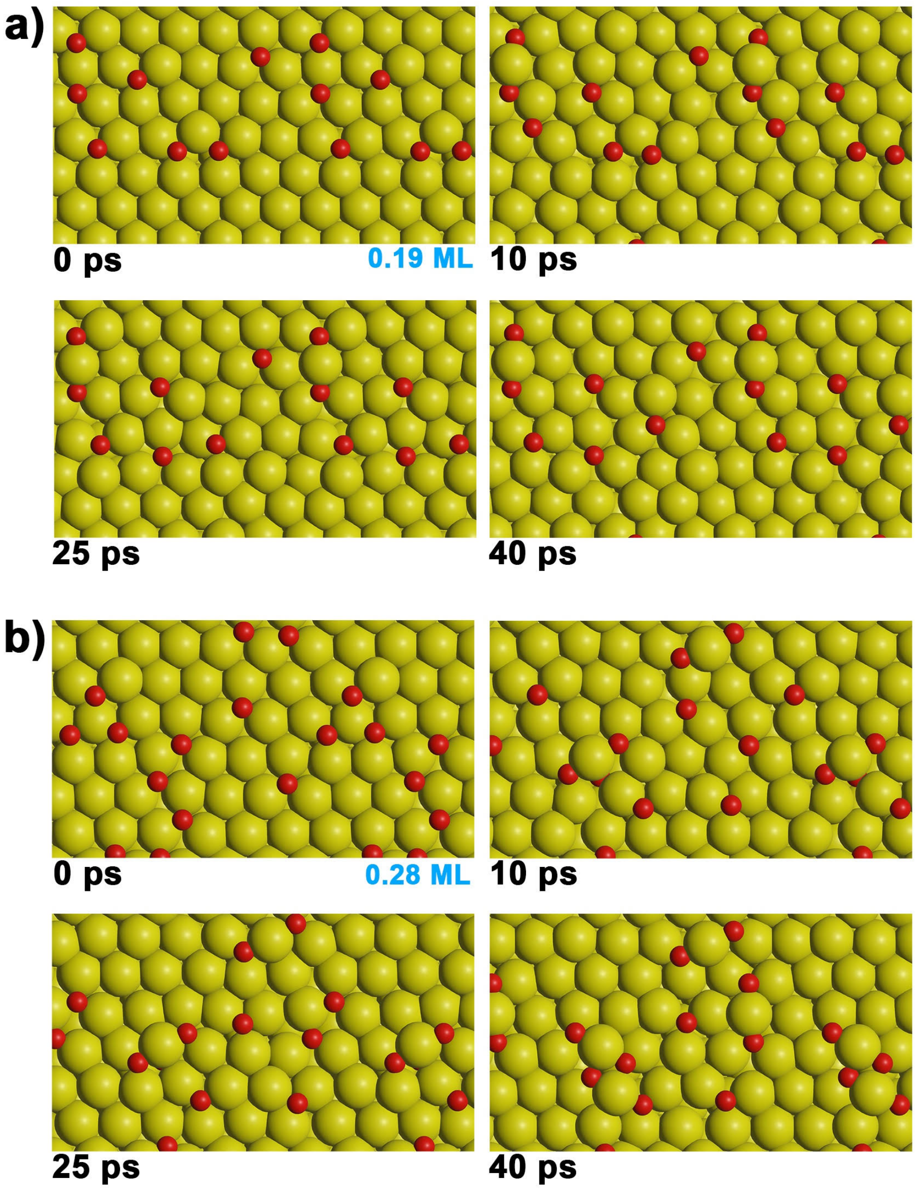


**Figure S9** Snapshots from AIMD simulations illustrating different degrees of surface restructuring induced by adsorbed atomic oxygen on flat Au(111). Surface evolution from 0 ps to 40 ps is shown for two different oxygen coverages, 0.19 ML (a) and 0.28 ML (b).

a) b)

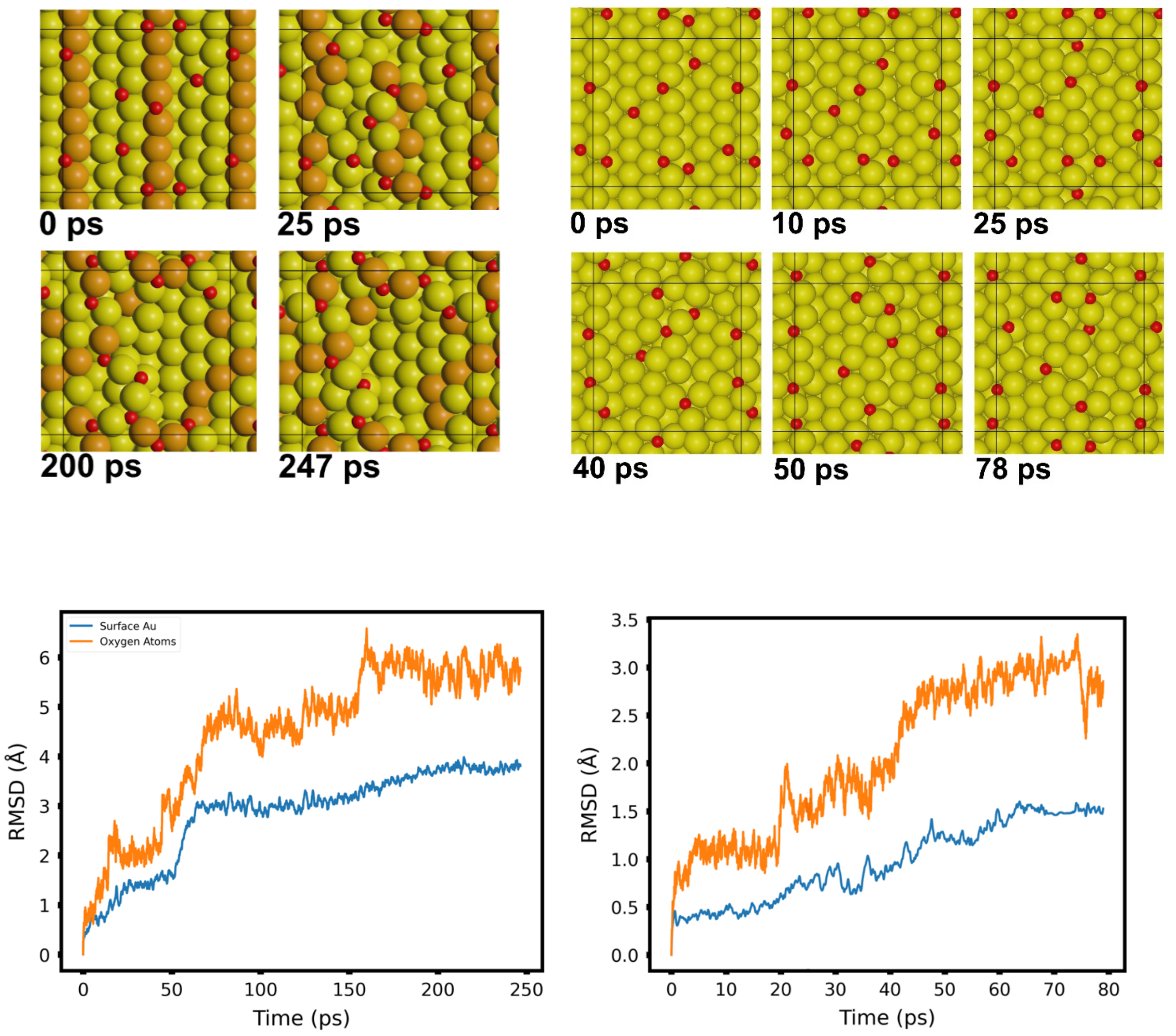


**Figure S10** Snapshots from AIMD simulations (top) and the corresponding RMSD plots (bottom) comparing the displacement of the Au surface and adsorbed O atoms for the stepped Au(221) surface (a) for the first (long) AIMD simulation. (The traces shown in Figure 5b are a subset of these traces for the initial 60 ps) and (b) for the second AIMD simulation with a different initial configuration of the oxygen atoms. Color coding: snapshots: Au, yellow or orange; O, red. Orange color indicates Au atoms originally at steps in (a); RMSD plots: O atoms, orange; surface Au atoms, blue

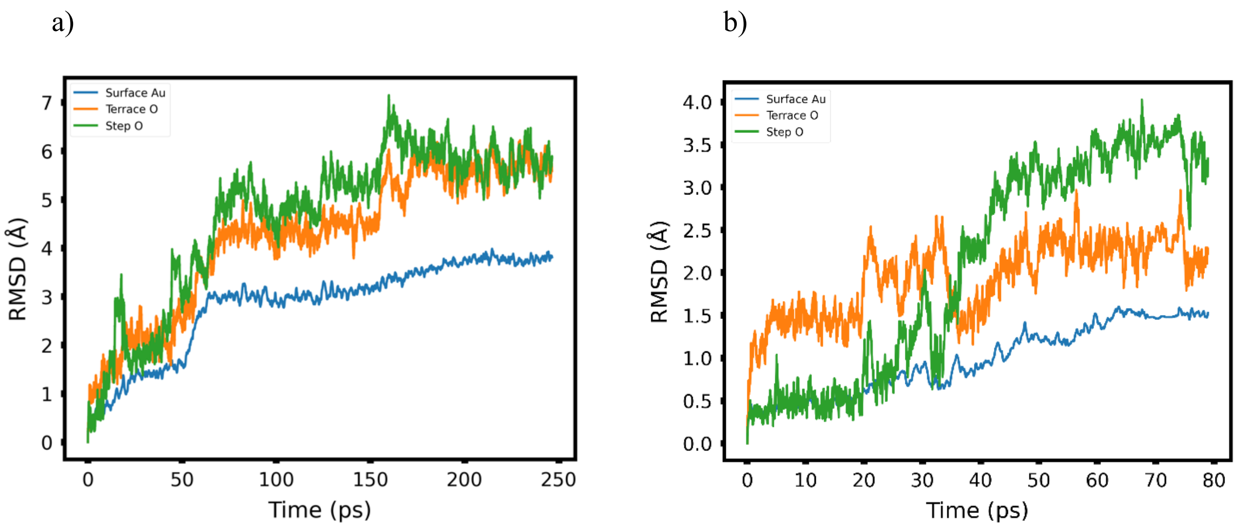


**Figure S11** RMSD plots for the stepped Au(221) surface analogous to Figure S10 but with the RMSD values of oxygen species evaluated individually for different types of initial adsorption sites (step or terrace). Oxygen atoms on terraces: orange; oxygen atoms on steps: green; surface Au atoms: blue.

**Discussion of the statistical significance of the AIMD simulations**

Figure S10a shows the RMSD traces for the entire simulation on the (221) surface presented in Figure 5b. The traces indicate that rearrangements on the surface occur throughout the entire simulation period. However, the extent of these rearrangements can vary significantly over different time intervals. For the trajectory presented here, a major rearrangement takes place within 10 ps between about 60 ps and 70 ps, followed by a period from 80 ps to 100 ps where little change in the RMSD values is observed. Statistically, the oxygen atoms have moved about one-third of the unit cell dimension, substantiating the conclusion that significant rearrangements involving essentially all of the surface O atoms are taking place. Although the Au atoms move by about half this length, the average movement is considerably larger than the distance between equivalent neighboring sites, indicating that most of the Au atoms are also changing their positions during the simulation.

It is clear that the details of the temporal evolution are subject to statistical fluctuations inherent to such simulations and depend on the initial configuration chosen for the trajectory. To this end, an additional 80 ps simulation was performed with a different initial configuration of the oxygen atoms (at the same coverage), where 75 % of the oxygen atoms were initially located at step edges. The corresponding RMSD trace (Figure S10b) shows an increase in RMSD values for both atom types over the 80 ps trajectory, consistent with the other simulation. Quantitatively, the RMDS values are generally smaller for the second run. However, the values at around 60 ps, where a large-scale rearrangement occurs in the first run, are very close in both cases. This indicates that the differences between the two runs are largely due to statistical

fluctuations expected for different trajectories. As the number of oxygen atoms located at or close to step sites was higher in the second run (6 out of 8 instead of 4 out of 8 in the long simulation), it is interesting to compare the RMSD values for the oxygen atoms on different initial adsorption sites separately for both simulations (Figure S11). This analysis provides no evidence for systematic difference in the dynamics of the two species, consistent with the interpretation that the observed differences in the details between the simulations are likely due to statistical fluctuations expected for different trajectories.
